# Single-Cell Analysis

**DOI:** 10.3390/cells9091993

**Published:** 2020-08-29

**Authors:** Tuhin Subhra Santra, Fan-Gang Tseng

**Affiliations:** 1 Department of Engineering Design, Indian Institute of Technology Madras, Tamil Nadu 600036, India; 2 Department of Engineering and System Science, National Tsing Hua University, Hsinchu 30013, Taiwan; fangang@ess.nthu.edu.tw

Cells are known to be the most fundamental building block of life. Cells form our bodies grow, reproduce, process information, and they can respond to the stimuli and process different chemical reactions [1]. However, to date, the interactions between cells, the responses of their organelles to molecules and intracellular behaviour remain unknown. For the understanding of cellular physiological interactions with different molecules and organelles, an average ensemble measurement of millions of cells together is not enough to provide detailed information about cells [2,3,4]. Again, bulk analysis of millions of cells cannot explore cellular heterogeneity characteristics and dynamics at the molecular level, in a particular cell populations [2,3,4]. However, the investigations on the progress of any disease have remained challenging because of the physiological states of cells and heterogeneity nature of cells in a specific given population [2,3,4,5]. Compared to the average analysis of millions of cells together in bulk, single-cell analysis (SCA), provides clear information about each cell, such as specific biological factors of cell, which is helpful to understand stem cells behaviour or tumour progression [2,3,4,5,6,7,8]. Moreover, SCA analysis can characterize in detail the molecular contents of cells, related to cell state and type, spatial and temporal transformations, and micro-environment. Single-cell omics such as genomics, proteomics, transcriptomics, metabolomics/fluxomics can promote unrevealed information in regards to the functional mutation and cells copy number variations [2,4,9,10,11]. All of these omics analyses may involve mathematical and computational modelling to investigate cellular functions ranging from the complete genome to living organisms. Single-cell genomics and gene expression profiling are used widely in medicine for optimizing several biological, biomedical, and pathological conditions for various disease analyses [2,9,10,11]. The single-cell proteomics and transcriptomics analyses play a significant role in the study of cellular heterogeneity characteristics, complex diseases such as cancer, and evolve the dynamics of differentiation and quantifying transcriptional stochasticity [2,4,10]. Moreover, these analyses help us to study some biological phenomena such as tissue regeneration, immune response and embryonic development. Single-cell RNA sequencing (scRNA-seq) can unlock the mystery of gene expression and track the trajectories of distinct cell lineages in development. Single-cell metabolomics can elucidate in detail about the phenotypical variations of cells [11]. Understanding of single-cell metabolic reactions across organelles helps us to identify any disease pathologies. It also can provide holistic and comprehensive insights at the cellular or even subcellular level to understand the cellular organism responses, under different pathophysiological conditions. Furthermore single-cell analysis helps us to understand cellular organelle responses to the environment and intracellular interactions at a more profound level than bulk analysis, which would accelerate the developments of therapeutics and diagnostic processes [2,3,4]. Due to the tremendous applications and prospects of single-cell analysis, the research activity and scientific publications are increasing day by day. Figure 1 shows the worldwide single-cell related scientific article publications and their various applications, respectively. From this figure, it is clear that the research activity increases 3 to 4-fold in the last five years compared to previous years. 

The last two decades have seen a rapid advancement of micro and nanotechnologies and their integration with life science, chemistry, chemical engineering, mechanical and biomedical engineering; the new era has started based on the development of bio-microfluidics or Bio-MEMS devices or µ-TAS (micro-total analysis systems). These devices have the ability to analyze single-cells in the micro or nanofluidic environment. Moreover, the devices not only detect and manipulate bio-samples in micro/nanofluidic environment but also they can consume a minimum amount of samples for in-detail analysis [2,3,4,7,8,12]. The recent development of micro/nanofluidic devices can easily detect and analyze different single-cell omics such as genomics, proteomics, metabolomics as well as analyzing the different levels of disease progression [5]. Again, the devices have the ability to do single-cell therapy, diagnostics [13] and their analysis by using different physical approaches such as electroporation [14,15,16,17,18], mechanoporation [19], photoporation [20,21,22,23], microinjection [24,25] etc. Besides the extensive applications for cell separation, isolation, manipulation, and lysis, these devices are potentially helpful for studying cellular biochemical, mechanical and electrical characterization at single-cell or in subcellular level [26]. The devices have the ability for single-cell sequencing, which is potentially useful to study cellular heterogeneity, molecular mapping, immune infiltration and epigenetic changes. Using these micro/nanofluidic technologies, the development of fluorescence-activated cell sorting (FACS) will potentially act as a rapid, reliable, and sensitive immunodiagnostic method in the clinical laboratory. It can analyze the content of the fluorophore and biomarkers at the single-cell or even subcellular level and sort them based on the desired phenotype. Nowadays, the multiplex biomarker analysis is of great interest using FACS, due to its isolation and identification capabilities of immune cells to a specific phenotype.

This Special Issue of *Cells* entitled “Single-Cell Analysis” covers the recent advancements of single-cell analysis using different approaches. Chio et al. [27] illustrated single-cell RNA sequencing (sc-RNA-seq) in combination with protein and DNA analysis. The proposed techniques help us to understand more details about cellular heterogeneity and biological information from a single-cell, which is unobtainable from bulk analysis. The authors reviewed the recent advancement of scRNA-seq profiles integrated along with proteomic or genomic data, which can reveal far better insights into the complex cellular heterogeneity characteristics than transcriptome measurements alone. They also briefly discussed scRNA-seq technology, together with conventional as well as advanced microfluidic approaches with their advantages, limitations, future prospects and potential biological and biomedical applications. The single nucleus RNA sequencing (snRNA-seq) of the whole mammalian heart was emphasized by Wolfien et al. [28], which allows us to explore cellular composition and cell features. The snRNA-seq quantification and clustering can convey precise cell types affirmation. Their study revealed 24 individual clusters with 25.3% fibroblasts, 28.8% endothelial, and 22.8% cardiomyocytes comprising the major cell populations. Again, they performed the RNA velocity analysis, which allows to emphasize the transcription kinetics and unlock the details of the dynamics as well as the identified cell clusters interconnectedness. They also identified the cardiomyocytes subgroups with distinct marker profiles. The quantification of mRNA study at single-cell level was illustrated by Jonasson et al. [29]. This quantification study can be widely applicable for cellular characterization, immune responses, and mapping of intra-tumoral heterogeneity etc. They developed an easy to use, fast and flexible process for total mRNA quantification within a single-cell, using global reverse transcription mimicked by quantitative PCR (polymer chain reaction). Moreover, the author performed human fibroblasts cells and three sarcoma cell line analyses, which disclosed the variations of cell type and total quantification of mRNA. The results showed that total mRNA levels among the cells have an eight-fold difference. The proposed method does not prescribe sequencing, although it can be effortlessly associated with global RNA sequencing and targeted qPCR and for additional cellular analysis.

The localized high-risk prostate cancer circulating tumour cells (CTC) genomic analysis was discussed by Rangel-Pozzo et al. [30]. The tumour heterogeneity is one of the major causes of failure in prostate cancer prediction and prognosis. The CTC analysis can provide individual patient-specific clinical assessment, due to unlocking the mystery of tumour-derived and germline-specific genetic information more precisely in comparison with a single diagnostic biopsy. In their study, the authors combined a filtration-based CTC isolation method with prostate cancer CTC immunophenotyping to identify the prostate cancer CTCs. They found that localized high-risk prostate cancer patient CTCs can provide a high number of telomere signals with lower signal intensities. Whole-exome sequencing of high-risk prostate cancer CTCs was carried out to gain information about cellular genetic diversity or heterogeneity. The authors concluded that single-cell genome sequencing (whole-exome sequencing and 3-D telomere profiling) approaches were useful for CTC heterogeneity characteristics in a treatment-naïve prostate cancer patient risk group and copy number alternation amplification, which visualized in a localized high-risk patient, could be playing a major role in the therapeutic resistance in prostate cancer. Hepatocyte cells are the primary parenchymal cells of the liver. These cells have a significant impact on liver homeostasis and disease process. Chang et al. [31] discuss the heterogeneity characteristics of mouse hepatocytes and their distinctive functions during cholestatic liver injury using single-cell transcriptome analysis. They reported single-cell RNA sequencing and mouse liver injury model induced by Bile duct ligation (BDL). For their study, the qPCR and Western blot were used for gene expression and immunofluorescence, to detect the gene expression of hepatocytes.The detection of distinctive hepatocyte cluster (BDL-6) expressed extracellular matrix gene, and it might be indicating hepatocytes undergo epithelial-mesenchymal transition. Moreover, hepatocytes of BDL-6 can perform angiogenesis during cholestatic injury as a function of tissue repair. Again, they reported BDL-2, BDL-3, BDL-4, and BDL-5 cholestatic hepatocytes clusters were engaged in the inflammatory process in different ways.

Liu et al. [32] proposed various methods of single-cell proteome analysis and their potential biomedical applications. The single-cell proteome analysis can help us to understand cellular heterogeneity characteristics, the molecular dynamics of the cell, as well as the clinical applications for tumour treatment and drug development. The authors initially discuss the conventional protein characterization and analysis techniques, together with enzyme-linked immunospot assay, fluorescence flow cytometry, mass cytometry, and capillary electrophoresis. Later they discuss single-cell protein analysis using advanced microfluidic approaches such as microfluidic fluorescent flow cytometry, microwell-based assay (micro engraving), droplet-based microfluidics, single-cell Western blotting, and microchamber-based assay (barcoding microchips). The advantage, limitations and future prospects of single-cell protein analysis are also elaborated. The extremely sensitive and multiplexed in-situ protein analysis was performed by using cleavable fluorescent streptavidin, and cleavable biotin-conjugated antibodies were discussed by Liao et al. [33]. Moreover, the author successfully quantifies proteins in formalin-fixed paraffin-embedded tissues. The in-situ protein analysis facilitates the understanding of normal physiological behaviour and disease pathogenesis. In their study, first, they recognized protein targets by the cleavable biotin-labelled antibodies and then used cleavable fluorescent streptavidin to stain the protein targets. The layer-by-layer signal amplification was performed using cleavable biotin-conjugated orthogonal antibodies and cleavable fluorescent streptavidin. As a result, the protein detection sensitivity enhanced at least 10-fold in comparison with the current available in-situ proteomic methods. This approach is applicable for the study of in-situ DNA, RNA and metabolic analysis. Yeh et al. [34] demonstrated the generation of a monoclonal cell line from single-cell cloning using a disposable microfluidic device. The generation of monoclonal cell lines is an important mean to produce proteins for basic biological research and therapeutic drug development purposes. In their approach, they reduced the development time to validate the generation of monoclonality of the cloned cells. Most of the monoclonal cell line generation took the longest time (approximately one month) with complex techniques. Here, the author produced monoclonal colonies of actin-GFP plasmid-transfected A549 cells using a simple disposable microfluidic device within nine days. Later this cell line was transferred to wells within plates to allow further expansion by using a tissue puncher.

Single-cell genomics and proteomics in integration with three-dimensional (3D) cell culture technique can execute the new prospects for the unveiling of tumour heterogeneity. Alföld et al. [35] distinguished the individuality of two-dimensional (2D), three-dimensional (3D) and in-vivo models by using multi-parametric single-cell mass cytometry technique. The lung cancer markers were characterized by using single-cell mass cytometry and compared with different in-vitro cell culture techniques. Their research showed that by using an organic scaffold, A549 lung cancer cells could grow, or scaffold-free 3D culturing techniques could express in-vivo tumours of gene and protein far better than standard 2D cultures technique. Moreover, the authors showed the rate of heterogeneity in 2D and 3D cultures and in solid-tumour. Lin et al. [5] investigated how single-cell technology has been applied to inspect several infectious diseases. They studied how cellular heterogeneity is linked substantially with the progression of infectious disease using fluorescence-activated cell sorting and next-generation sequencing. The genomic and phenotypic biomarker characterization, as well as host-pathogen interaction at the single-cell level, can help us to understand the unknown infection mechanisms and potential diseases treatment. A piece of detailed single-cell information from primary cells can identify rare but important cell subtypes, which help us to understand the complex interplay between cells and the immune system. The advancement of single-cell analysis also can help us for the development of drugs, vaccines and potential biomarkers.

Gupta et al. [36] reviewed various single-neuron models, single-neuron behaviour, and their analysis. In comparison with the bulk analysis of millions of neurons together, the single-neuron analysis can emphasize the detail of pathophysiology, electrophysiology, anatomical differences, structural and functional features etc., at the single-neuron or even sub-neuron level. The single-neuron analysis can play an important role in studying the in-between communications of neurons and provide essential information of the brain activity. The author described the details of the single-neuron mapping and the electrophysiological recording. They have highlighted the recent advancement of single-neuron manipulation, isolation, and therapy using microfluidic devices following different physical approaches such as electroporation, microinjection, microelectrode array, optogenetic techniques, optical transfection, etc. Finally, the authors added the impact of artificial intelligence in single-neuron and concluded the limitations and future prospects of single-neuron analyses.

In conclusion, this Special Issue of *Cells* emphasizes in detail about single-cell analysis using micro/nanofluidic devices, capillary electrophoresis, fluorescence-activated cell sorting (FACS), single-cell Western blotting, single-cell mass cytometry, enzyme-linked immunospot assay study, quantitative polymerase chain Reaction (qPCR) technique and next-generation sequencing (NGS) etc. The single-cell RNA sequencing (scRNA-seq) technologies were briefly reviewed by using microfluidic devices to understand cellular heterogeneity characteristics. The single nucleus RNA sequencing (snRNA-seq) was used to investigate the cellular composition and cell features of an entire adult mammalian heart. The mRNA levels in single-cell are quantified using global reverse transcription method followed by quantitative PCR. This quantification technique can help us to characterize cell molecules for different types and states of cells with the temporal variation of the microenvironment. The single-cell transcriptome and analysis were performed using Western blot, qPCR as well as immunofluorescence assay. The advancement of microfluidic technologies such as microfluidic fluorescent flow cytometry, microwell-based assay (micro engraving), droplet-based microfluidics, micro-chamber based assay (barcoding microchips), are used for protein analysis of single-cells. The cleavable biotin-conjugated antibodies, as well as cleavable fluorescent streptavidin (CFS), are employed for delicate in-situ protein analysis. The microfluidic single-cell cloning device is used to generate monoclonal cells for producing proteins with high reproducibility, and it can be applied for the production of therapeutic drugs. The single-cell mass cytometry was employed for lung cancer markers characterization and comparing the results with in-vitro 2D and 3D cell culture methods for drug screening applications. The importance of single-cell technologies has been well emphasized, in particular, for controlling and investigating infectious diseases. Finally, the behaviour of single-neuron and their analyses are discussed in detail by using single-neuron models, single-neuron mapping and electrophysiological recording, single-neuron isolation, manipulation and therapy. Further, the research area of artificial intelligence concerning single-neurons is highlighted.

## Figures and Tables

**Figure 1:**
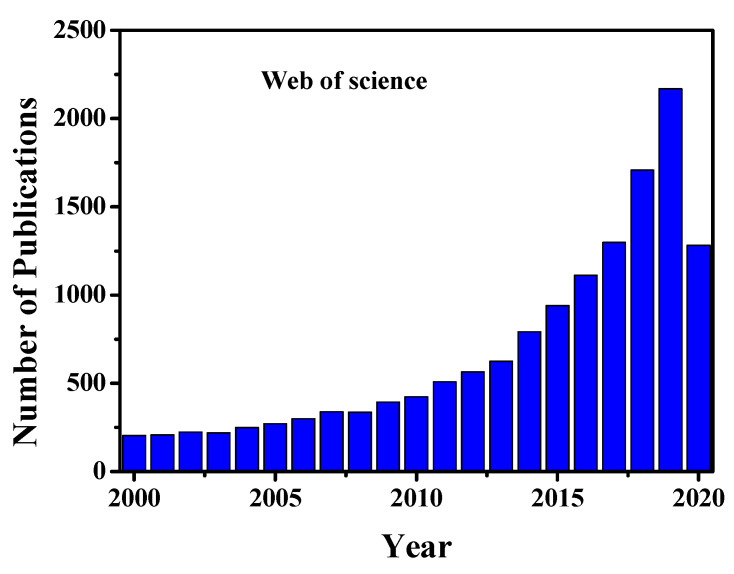
Year wise single-cell related publications. The data are adapted from web of science. Data until 25th August 2020.
